# Rekindling the Relevance of Obstetrical Transvaginal POCUS: Overcoming Barriers to Ensure Patient-Centered Care

**DOI:** 10.24908/pocus.v8i2.16855

**Published:** 2023-11-27

**Authors:** Alexis Salerno, Resa E Lewiss

**Affiliations:** 1 Assistant Professor of Emergency Medicine, Department of Emergency Medicine, University of Maryland School of Medicine; 2 Professor of Emergency Medicine, The University of Alabama at Birmingham

**Keywords:** Emergency medicine, transvaginal ultrasound, first trimester, Medical Education, Point of Care Ultrasound(POCUS)

## Letter

The transvaginal pelvic point of care ultrasound (POCUS) examination remains a patient-centered and relevant examination. Since 2008, emergency medicine physicians are required to learn, perform, and interpret POCUS examinations to deliver safe and patient-centered diagnostic and procedural care. Pelvic POCUS is one of these core applications in the emergency physician scope of practice. A pelvic POCUS examination seeks to answer the focused question, “Is there an intrauterine pregnancy (IUP)” and risk stratifies the patient when ectopic pregnancy is a clinical concern 1. Transvaginal ultrasound (TVUS) is necessary beyond transabdominal POCUS as it enhances the image quality of the uterus, ovaries and adnexal complexes. It can also assist providers in obtaining further clinically important information. 

 In a 2006 survey, 25% of community-based emergency departments reported having neither an ultrasound technologist available in the hospital at night nor a radiologist available to read the examinations performed 2. Consequently, most emergency physicians are responsible for and should be able to execute a TVUS examination for safe patient care. Not making a timely diagnosis in a patient with symptoms suggestive of an ectopic pregnancy can be life-threatening 3.The pelvic POCUS examination is especially relevant to emergency physicians who practice outside of a tertiary academic medical center and without 24-hour radiology-based ultrasound services. This represents the majority of emergency physicians in practice. A 2017 survey of emergency physicians who completed the 2017 ConCert examination found that 76.3% of participants identified as community emergency physicians, while 19.6% identified as academic emergency physicians 4.

Despite the established role of the pelvic POCUS examination in emergency medicine patient care practice, we are increasingly concerned that residents are not being taught the TVUS technique and faculty are not using this diagnostic imaging test for patient-centered care. A 2020 Society for Clinical Ultrasound Fellowships survey asked directors to report on the use of TVUS by faculty, fellows, and residents: Shockingly, only 20% of emergency physicians used TVUS regularly. 58% reported using TVUS occasionally 5. 

We believe there are multiple factors contributing to what we perceive as a decline in performing this examination. 1) Better POCUS hardware: there is an improved quality in transabdominal imaging making TVUS considered less necessary. 2) Few training opportunities: emergency medicine residents find limited training opportunities when working with attendings who do not perform TVUS and when patients are preferentially transported to the radiology for TVUS. 3) Infection control measures: POCUS leaders anecdotally note the increasing surveillance in transducer cleaning and sterilization practices as a deterrent to performing a TVUS examination 6.

## TVUS Still Offers Improved Imaging

We believe that TVUS continues to be relevant, important, and patient-centered to emergency medicine practice. The TVUS examination offers improved image quality over the transabdominal technique. The TVUS examination identifies early pregnancy structures one week earlier than a transabdominal examination with the use of a curvilinear transducer in a patient with a full bladder 7. Logistically, the TVUS transducer is higher frequency. We acknowledge that ultrasound imaging has improved over the last decade, and in some instances the high frequency linear transducer can identify an IUP 8;however, transabdominal imaging is not equivalent to TVUS. One cohort study of over 500 patients showed that EP performed TVUS helped diagnose a viable IUP in 50% of patients with an inconclusive curvilinear transabdominal ultrasound 9. As a result, the ED length of stay was 3 hours for patients with emergency physician performed TVUS examination, versus 6 hours for patients with a technician in radiology performed TVUS examination 9. The image quality improvement by TVUS may be greater in patients with high BMI, abdominal surgical scars, or poor visualization of structures due to bowel gas.

## TVUS is a Teachable Skill

With increased experience and training in TVUS, emergency physicians can determine the presence or absence of an IUP. One study showed that emergency medicine residents require a relatively short training period to learn and competently perform a TVUS 10. After a 1-hour didactic session, a written examination, and 10 supervised studies, the residents were able to perform a TVUS examination to evaluate for IUP with good concordance with the ED director of ultrasound 10. Learners continued to benefit from performing a greater number of TVUS exams and felt confident after performing 25 examinations 11. 

To help medical educators ensure that TVUS remains a procedure that residents learn, we suggest creating training opportunities. The opportunities to learn TVUS are many: direct patient care in the emergency department, a rotation on the obstetrics-gynecology service, a rotation in radiology, and of course the emergency medicine POCUS rotation. Simulation centers that offer both static and dynamic pelvic ultrasound simulators are excellent education resources. Simulated examinations offer many advantages, including competency assessments and the opportunity for direct feedback 12. One study showed that simulation based TVUS training not only increased clinician comfort in performing the examination and decreased the duration of live TVUS examinations, but also showed that residents who had simulation training had decreased patient discomfort scores 12. 

## TVUS Transducers Can Be Maintained

There has been an increased focus on infection control guidelines by national organizations, such as the Joint Commission on Accreditation of Healthcare Organizations (JCAHO), specifically on the use of TVUS. We believe that if radiology and gynecology staff are able to follow the cleaning protocol, then certainly the emergency department can too. Cleaning and disinfecting the TVUS requires high level disinfection (HLD) 13. We acknowledge that the education and maintenance for the TVUS transducer may be perceived as cumbersome and difficult to maintain, yet there are many solutions. This could be performed in the emergency department where team members learn the HLD protocols. This requires upfront resource investment and then the process should be easy to follow 14. Alternatively, some EDs, which are unable to support a HLD system can share resources with the Department of Radiology or the hospital central sterilization department. Further research on cost/resource utilization of the TVUS may help overcome this barrier. 

In conclusion, the pelvic TVUS ultrasound examination is an easy to learn, patient-centered examination. While TVUS is being infrequently taught and performed, its significant benefits should prompt research into how this technique can be incorporated into bedside practice.

## Disclosures

This work has not been presented at meetings, no grant support was received. REL serves on the Medical Advisory Board for EchoNous, on the board of PURE, on the board of Society for Clinical Ultrasound Fellowships (SCUF), and previously received equipment support from Phillips Healthcare and Butterfly Network. Otherwise, there are no disclosures of relevant commercial interests. 
